# Descriptive study of burnout, compassion fatigue and compassion satisfaction in undergraduate nursing students at a tertiary education institution in KwaZulu-Natal

**DOI:** 10.4102/curationis.v40i1.1784

**Published:** 2017-09-22

**Authors:** Christina T. Mathias, Dorien L. Wentzel

**Affiliations:** 1 School of Nursing & Public Health, University of KwaZulu-Natal, South Africa; 2 Office of Rector, North West University, South Africa

## Abstract

**Background:**

Studies have investigated burnout and compassion fatigue among nurses and effects in the nursing profession. However, there are limited investigations of burnout and compassion fatigue among undergraduate nursing students in South Africa, as nursing students may experience distressful situations during their nursing education course, which may have an impact during their training and in their profession as they graduate.

**Purpose:**

The purpose of this descriptive study was to describe compassion satisfaction, compassion fatigue and burnout among undergraduate nursing students at a tertiary nursing institution.

**Methods:**

A quantitative descriptive study was conducted to describe compassion satisfaction, compassion fatigue and burnout among undergraduate nursing students at a tertiary nursing institution in KwaZulu-Natal. Convenience sampling was used.

**Results:**

Sixty-seven undergraduate students (26 third-year and 41 fourth-year nursing students) took the self-test Professional Quality of Life Scale (ProQOL). The study results indicate that undergraduate students experienced average levels of compassion fatigue, burnout and compassion satisfaction.

**Conclusion:**

As shown in the study, some of the undergraduate students are experiencing compassion fatigue and burnout, associated with relieving suffering of others. Therefore, knowledge of compassion fatigue and burnout and the coping strategies should be part of nursing training.

## Background of the study

The backbone of a true caring professional is compassion, where care providers have a feeling of empathy for the suffering or misfortune of others and understand the client’s personal feelings or experiences without being judgemental (Williams & Stickley 2010:73). Therefore, providing quality nursing care goes together with empathy, which allows the nurse or student nurse to care for the patient in the manner he or she would want to be cared for if he or she were in the same situation; this brings about compassion, knowing the suffering of others and wanting to relieve it (Radley & Figley 2007:210). Student nurses empathise with the patients’ suffering in order for them to provide quality care; hence, they are at the edge of experiencing secondary trauma as they are witnesses to their patients’ suffering (Figley 2002:1435; Stamm 2010:13).

Beddoe and Murphy (2004:307), Michalec, Diefenbeck and Mahoney (2013:316) and Marques da Silva et al. (2014:1) explain that nursing education is designed to provide a platform for fostering empathy and compassion in the nursing student as a way of preparing the student nurses to be professionals to care for others. When compassion has been demonstrated for a long time, compassion fatigue could manifest as a negative outcome. Compassion fatigue is described as being preoccupied with or re-experiencing the patient’s traumatic events thus reducing the caregivers’ aptitude or interest in bearing the suffering of other people (Figley 2002:1435). The nature of nursing potentially diminishes one’s emotional responsiveness because of exposure to stressful situations, personal, professional ethical conflicts and dilemma, frequent contact with dying patients and suffering and fear of contracting infections (Tomaschewski-Barlem et al. 2014:80). As a result, compassion fatigue subjects nurses to be ineffective or inefficient in their duties, in caring for patients. Hence, the translation of theoretical knowledge into practice is affected as the student nurse avoids performing the skills that will remind him or her of the traumatic experiences he or she encountered earlier (Alkema, Linton & Davies 2008:104; Figley 2002:1438).

There is a current interest in traumatisation experienced by healthcare workers; however, there appears to be discord as to the formal definitions of burnout, secondary stress syndrome, secondary stress in traumatology, secondary victimisation, secondary traumatic stress, secondary survivor, compassion fatigue and vicarious traumatisation (Coetzee & Klopper 2010:241). Thus, subsequently the terms being used interchangeably lead to confusion and management of the condition (Coetzee & Klopper 2010:235).

Burnout is ‘associated with feelings of hopelessness and difficulties in dealing with work or in doing your job effectively’ (Stamm 2010:13). Burnout can be further defined as ‘a syndrome composed of emotional exhaustion, depersonalization, and reduction of personal accomplishments’ (Stamm 2010:13). When burnout occurs, the physical and mental capacity of the caregiver is affected which brings a feeling of negative self-concept and attitudes towards work; enthusiasm and vigour of working diminishes, hence compromises delivery of quality nursing care (Keidel 2002:200; Stamm 2010:13).

Despite nurses displaying compassion fatigue, as a negative outcome of long-term experience of empathy for traumatised patients or clients, compassion satisfaction can also result, which is a feeling of pleasure that is derived from ably discharging one’s duties (Alkema et al. 2008:104; Stamm 2010:12).

Maximisation of nursing productivity, job satisfaction and efficiency in the undergraduate nursing students depends on prevention or minimisation of burnout and compassion fatigue during their training. Burnout or compassion fatigue among nursing students could enable insufficient attainment of knowledge and skills among students, thus affecting the quality of care, which could expose patients to care-related risks (Marques da Silva et al. 2014:2; Rudman & Gustavsson 2012:999).

The conceptual framework used to guide the study was The Compassion Fatigue Process (Figley 2001:3). This process highlights that empathy is the cornerstone and the motivator of caring, and when it is coupled with emotional energy, it sustains a therapeutic nurse–patient relationship and promotes delivery of targeted quality patient care. The Compassion Fatigue Process by Figley (2001:5) is the model that explains 11 variables for compassion fatigue to manifest, with empathy being the fundamental for compassion satisfaction and compassion fatigue.

Nursing students interact with dying patients, watching some of them die and experiencing guilt concerning their deaths. Other stresses that nursing students encounter could include academic studies and finances, and they are also insecure about their clinical competence, which can cause compassion fatigue in nursing students (Parkes [1985] cited in Liora Kordero & Livia 2014). Michalec (2013) reiterates undergraduate nursing students are exposed to stressful situations especially those in more clinical-oriented years of the training.

### Problem statement

Michalec et al. (2013:314) endorse that ‘burnout and compassion fatigue negatively impact nurses’ well-being, job satisfaction, and willingness to remain in the profession’. The issues that predispose nurses in hospital settings to burnout and compassion fatigue are the same issues undergraduate nursing students face during their clinical placement. Hence, nursing students are also at risk of developing burnout and compassion fatigue during their nursing training. Tomaschewski-Barlem et al. (2014:81) recognise burnout syndrome in the context of students as the feeling of tiredness because of the high demands of study, scepticism and a distant attitude in relation to studies and the perception of being ineffective students.

Rudman and Gustavsson (2012:998) conclude there is intent to leave the profession by some of the undergraduate nursing students as they experience exhaustion or disengagement because of stress in relation to the academic and the clinical components of the nursing programme. Therefore, it is important to assess stress in undergraduate nursing students and to increase their knowledge of burnout and compassion fatigue, in order to enhance their training by promoting academic performance and nursing skills and hence provision of quality patient’s care (Marques da Silva et al. 2014:2).

Stamm (2010:13) describes compassion satisfaction as gratification obtained when doing one’s work to the best of one’s ability. Stamm (2010:13) reinforces that compassion satisfaction is the feeling of pleasure that results from ably executing one’s duties. When a student nurse capably provides care that contributes to improved well-being of the patient, he or she feels happy and content with the knowledge and skills that he or she is attaining in his or her nursing education. This in turn gives him or her increased morale to work harder so that he or she should continue providing quality nursing care to the patients he or she is going to care for. Therefore, it is important to know what causes compassion satisfaction so we are able to learn more about how to promote compassion satisfaction.

### Purpose of the study

The purpose of this descriptive study was to describe burnout, compassion fatigue and compassion satisfaction in undergraduate nursing students at a tertiary nursing institution.

### Definition of terms

#### Burnout

Jenaro, Flores and Arias (2007:80) explain burnout as ‘a syndrome composed of emotional exhaustion, depersonalization, and reduction of personal accomplishments and is displayed as feelings of hopelessness, difficulties in dealing with work, and poor work performance’.

#### Compassion fatigue

Figley (2002:1435) describes compassion fatigue as being preoccupied with or re-experiencing the patient’s traumatic events, thus reducing the caregivers’ aptitude or interest in bearing the suffering of other people.

#### Compassion satisfaction

Stamm (2010:13) describes compassion satisfaction as gratification obtained when doing one’s work to the best of one’s ability.

#### Undergraduate nursing student

Piero (2011:1) describes an undergraduate as a person who is studying after post-secondary education or high school after meeting the tertiary institution’s entry requirements. In this study, third- and fourth-year undergraduate nursing students were used.

## Research design

A quantitative, descriptive design was used in this study.

### Setting

The study focused on undergraduate nursing students at the University of KwaZulu-Natal.

### Population, sample and sampling

Student nurses who were registered for the 4-year degree programme for the education and training as a nurse formed the target population. Third- and fourth-year nursing students were targeted as at these levels, students would have the highest clinical exposure and academic qualification thus be able to self-reflect on experiences. These years of study were chosen because their duration of possible exposure to stressful situation is longer than the first- and second-year undergraduate students. Tomaschewski-Barlem et al. (2014:81) emphasise undergraduate nursing students who are in their final year of study, advanced grades, are prone to burnout syndrome because of increased level of activities and requirements besides studying for examinations. The sampling frame was obtained from the class registers.

### Data collection tool

Section A of the data collection tool contains demographic data of the participants.

Section B of the data collection instrument comprises a self-test on Professional Quality of Life Scale (ProQOL). Stamm (2010:13) developed ProQOL which measures aspects related to caregiving professionals’ quality of life. The instrument consists of three sub-scales, compassion satisfaction, burnout and compassion fatigue, and comprises 30 questions answered per Likert scale.

The sum of the score on each research question was analysed as follows: When a participant attains a sum of 22 or less, this translates to low level compassion fatigue or compassion satisfaction or burnout. A sum between 23 and 41 translates to average compassion fatigue or compassion satisfaction or burnout. A sum of 42 or more can be interpreted as a high level compassion fatigue or compassion satisfaction or burnout.

The participants had indicated different languages; nevertheless, they were all able to write and speak English, evidenced by the course being offered in English and the research questionnaire being administered in English. As such, all the participants speak and understand English.

### Data collection process

After obtaining full ethical approval from the university, permission was obtained from the academic leader to conduct research with the third- and fourth-year undergraduate nursing students. Data were collected over the month of October 2015, and the researcher arranged classrooms at times that were easily accessible to the participants. Questionnaires were distributed over a month (October 2015) by the researcher, which took approximately 15 min to fill in. Introduction to the research was done by the researcher, not known to the students, who then solicited for participation in the research. Willing participants completed a written consent and then were given a copy of the questionnaire to complete. All questionnaires that were filled in were put into an envelope, which was sealed and taken for analysis by the researcher.

### Data analysis

Data were analysed using descriptive data analysis using the Statistical Package for Social Sciences (SPSS), version 23, with the assistance of the statistician. Descriptive statistics were utilised.

### Data management

The data obtained from the research conducted were kept by the research supervisor who is keeping the filled questionnaires in her lockable room. During the study, the data were stored on the researcher’s computer, access to which is restricted by a code only known to the researcher. All data will be kept for 6 years, after which they will be destroyed or for 2 years after publication of the research and then destroyed.

### Validity and reliability

The researcher ran a pilot test with a small set of participants from the study target population. The researcher did not involve in the main study those who participated in the pilot study, to avoid chances of changing the responses because of memorisation of the questions.

The perceived threat to external validity is the Hawthorne effect. It affects the finding of the study when the participants are aware that they are being observed especially if there is use of unusual apparatus to collect data (Burns & Grove 2001:41). In the study, the researcher used a simple and elaborative questionnaire, which does not need the participants to be observed when they are providing answers, and it is not an invasive tool that causes harm to their lives.

Internal consistency of ProQOL and the Cronbach’s alpha reliabilities for the scales are as follows: compassion satisfaction alpha = 0.87, burnout alpha = 0.90 and compassion fatigue alpha = 0.87. More than 200 peer-reviewed articles have used the tool. Therefore, the ProQOL manual is well established and valid, and it is reliable to be used (Stamm 2010:13).

### Ethical consideration

Full ethical permission was granted by the tertiary institution HSS/1235/015M. Participants were asked to give their consent, participation was voluntary and participants were assured that they could withdraw from the study at any time without being penalised Confidentiality was assured by securing safely all data obtained to which only the researcher and supervisor had access. Anonymity was maintained by numbering the participants. The participants were assured of their privacy because neither the researcher nor the research supervisor would link the participant to data provided because of the anonymous nature of the data collection tool. For easy analysis of data, the researcher used codes that were not linked to the participants. Because the researcher had no identities of the participants, the participants were not worried about their personal information appearing in the results of the study.

During the research process, the researcher monitored and observed the participants’ emotional and mental health; those who could have been affected emotionally during the data collection process would have been cared for by referring them to student counselling services.

## Results

Sixty-seven undergraduate Bachelor of Nursing students from the university participated in the research study. Twenty-six (39%) participants were third-year undergraduate students and 41 (61%) participants were fourth-year undergraduate students who took part in the study, totalling 67 participants. Population was 85, and accessed population or response rate was 67 (79%).

### Demographics

The participants’ ages ranged between 20 and 35 years; 54 (81%) of the participants were female and 13 (19%) were male. The majority of the participants were African, 57 (85%), the remaining Indian, 5 (7%), and other ethnicities, 5 (7%). The predominating language was isiZulu 42 (63%), English 21 (31%) and Afrikaans 1 (1.5%) (see Table 1).

**TABLE 1:** Demographic profile (*n* = 67).

Variable	Demographic profile	Frequency	Percentage
Age	20–24	57	85.07
	25–29	5	7.46
	30–34	4	5.97
	> 35	1	1.49
Gender	Female	54	80.59
	Male	13	19.41
Home language	English	21	31.34
	IsiZulu	42	62.69
	Afrikaans	1	1.49
	Other	3	4.48
Ethnicity	African	57	85.07
	Indian	5	7.46
	Other	5	7.46

*Source:* Authors’ own work

The number of participants (*N* = 67) and how they scored on each ProQOL Scale on the self-test are summarised in Table 2.

**TABLE 2:** ProQOL (*n* = 67) demonstrating low or average and high level scores attained by participants.

ProQOL Scale	Low compassion (*n* = 67) < 22	Average compassion (*n* = 67) 24–39	High compassion (*n* = 67) > 40
Compassion satisfaction	2 (3%)	64 (95.5%)	1 (1.5%)
Burnout	4 (6 %)	63 (94%)	0 (0%)
Compassion fatigue	36 (53.7%)	30 (44.7%)	1(1.5%)

*Source:* Authors’ own work

ProQOL, Professional Quality of Life.

### Burnout

According to the ProQOL Scale, 4 (6%) participants attained a low level of burnout, 63 (94%) participants attained an average level of burnout and no participants attained a score for high burnout.

### Compassion fatigue

In relation to ProQOL Scale, 36 (53.7%) participants attained a low level of compassion fatigue, 30 (44.7%) participants attained an average level of compassion fatigue and 1 (1.5%) participant attained a high level of compassion fatigue.

### Compassion satisfaction

In relation to the ProQOL Scale, 2 (3%) participants attained a low level of compassion satisfaction, 64 (95.5%) participants attained an average level of compassion satisfaction and 1 (1.5%) participant attained a high level of compassion satisfaction.

## Discussion

Undergraduate nursing students frequently encounter stress during their academic years. Stresses of academic workload, long clinical hours, mastering clinical skills, coping with emergencies, fear of making mistakes, traumatic experiences with death and negative encounters with patients can take a physical and emotional toll on students. Rudman and Gustavsson (2012:989) report that stress levels increased significantly throughout the years of the undergraduate programme.

Protracted exposure to stress can result in burnout, which can be because of ongoing exhaustion of the individuals’ internal drive (Rudman & Gustavsson 2012:989). Nurses suffering from burnout and compassion fatigue can affect negatively on care rendered to their patients and patient satisfaction (Michalec et al. 2013:314).

This study revealed that third- and fourth-year undergraduate students experienced average levels of compassion fatigue, burnout and compassion satisfaction.

### Burnout

Studies conducted with Swedish nursing students demonstrated an increase in burnout over the 3 years, from 31% to 41%. An increase in depressive mood and less fulfilment with life can be related to burnout. In contrast, Marques da Silva et al. (2014:4) describe that nursing students were experiencing a lower prevalence of burnout. Marques da Silva et al. (2014:5) postulated that nursing students were able to recognise and cope with stresses that they encountered.

Tomaschewski-Barlem et al. (2014:84) reinforce there is a mutual relationship between burnout and time spent with patients. It is evident in the results that the participants were involved in hands-on activities for 3–4 years; hence, they were exposed to stressful situations. As a result, students are physically, emotionally and mentally exhausted because of involvement in emotionally demanding situations (Benson & Magraith 2005:497). Marques da Silva et al. (2014:1) concur that stress because of academic environment may provoke burnout in undergraduate nursing students. This is evident in the time data were gathered; the undergraduate students were busy studying for end of year examinations, which is an obvious stressor in the students, hence the probability for average level of burnout.

### Compassion fatigue

Michalec et al. (2013:18) discuss that in third and fourth year of study, students have increased clinical exposure, thus providing more opportunity for hands-on opportunities with patients. The experience with patients further supported the students’ satisfaction and interest in their choice of profession; this resonates with the study findings as majority of the respondents reported average levels of compassion fatigue. The experience appeared to lessen burnout and compassion fatigue. Despite students’ role of performing clinical duties, students are protected, as the complete responsibility of patients’ care falls under the professional nurses’ responsibility. This ‘safety net’ could also defend the student from burnout and compassion fatigue (Michalec et al. 2013:19).

### Compassion satisfaction

Tomaschewski-Barlem et al. (2014:84) identified undergraduate nursing students who do not choose nursing as their first course have a sense of dissatisfaction in the course, and, as a result, they may manifest with signs and symptoms of wear out as they perform nursing activities; this may make them leave the profession. Therefore, it is presumed that 1.5% of the participants who displayed compassion satisfaction had nursing as his or her first choice of study. That could be a possible reason he or she finds pleasure in what he or she does.

Michalec et al. (2013:314) reinforce that because of the increase in clinical exposure in third and fourth year, this exposure may increase clinical competence and confidence, which may assist in promoting compassion satisfaction and lessening compassion fatigue and burnout.

Gibbons (2010:1300) further explains course organisation and emotional exhaustion also bring dissatisfaction in the undergraduate nursing student. The data were collected at the time students were at the end of their academic year and they were preparing for exams; as such they were emotionally and academically exhausted, hence the dissatisfaction in the majority (98.5%) of the participants.

### Limitations of the study

Sample size was small and only third- and fourth-year undergraduate nursing students took part in the study. Therefore, because of the small sample size used in this study the results will not be generalised. One setting was used which is also a limitation. Data collection was carried out at the end of the year, and thus the pending examinations taking place at the end of the year may have influenced their results.

### Recommendations

The study results provide evidence-based information that compassion fatigue really exists in the nursing profession, as nursing students are constantly exposed to stressful events during nursing education training and provision of patient care. Therefore, strategies to prevent and manage compassion fatigue need to be strengthened in nursing education, nursing practice and administration and further studies should be conducted. Pertaining to the study results, the following are the recommendations:

Nursing education: Although this study did not demonstrate that nursing students showed high levels of burnout and compassion fatigue, the researchers strongly advise that nursing students should be monitored for burnout and compassion fatigue. Knowledge of burnout and compassion fatigue, signs and symptoms and its coping skills and management should be incorporated in the undergraduate curriculum (Keidel 2002:205; Marques da Silva 2014:5).

Nursing practice: Nurse professionals should be helping student nurses from developing compassion fatigue and burnout by counselling them whenever they are faced with stressful situations. Nurse professionals should also provide feedback to nursing training institutions on how the students are doing in terms of empathy and compassion. Without feedback the college lecturers and authorities would not know if some students are developing burnout or compassion fatigue (Marques da Silva 2014:5).

Administratively: The institution should have a councillor to help those that exhibits signs of burnout and compassion fatigue and manage them in their early stages, and also to implement strategies that will help to minimise emotional and physical exhaustion (Coetzee & Klopper 2010:241)

In order to offer certainty of existence of compassion fatigue and burnout among undergraduate students, there is need for further research study on the same topic but including all the first-, second-, third- and fourth-year undergraduate students and also involving at least two institutions.

Another study could be a comparison study of the extent of compassion fatigue and burnout among the third- and fourth-year undergraduate nursing students with the goal of finding out if long exposure to nursing-related stressors facilitates development of compassion fatigue and burnout.

## Conclusion

From this study, it is evident that compassion fatigue and burnout exist in the nursing profession in particular during nursing education training; this is because of the nature of the nursing education course, which demands direct care action that exposes students to frequent contact with pain and suffering. As shown in this study, some of the undergraduate students are experiencing an average level of compassion fatigue and burnout. It is recommended that knowledge of burnout and compassion fatigue and its coping strategies should be part of nursing training.
